# Pain Reduction in Adults with Limb Spasticity Following Treatment with IncobotulinumtoxinA: A Pooled Analysis

**DOI:** 10.3390/toxins13120887

**Published:** 2021-12-11

**Authors:** Jörg Wissel, Alexandre Camões-Barbosa, Georg Comes, Michael Althaus, Astrid Scheschonka, David M. Simpson

**Affiliations:** 1 Department of Neurorehabilitation and Physical Therapy, Vivantes Hospital Spandau, 13585 Berlin, Germany; 2 Centro Hospitalar Universitário de Lisboa Central, 1169-056 Lisbon, Portugal; alexandre.barbosa@CHLC.min-saude.pt; 3 Merz Pharmaceuticals GmbH, D-60318 Frankfurt am Main, Germany; Georg.Comes@merz.de (G.C.); Michael.Althaus@merz.de (M.A.); Astrid.Scheschonka@merz.de (A.S.); 4 Icahn School of Medicine at Mount Sinai, Department of Neurology, New York, NY 10029, USA; david.simpson@mssm.edu

**Keywords:** limb spasticity, botulinum toxin type A, incobotulinumtoxinA, pain, pooled analysis

## Abstract

Some studies have shown that incobotulinumtoxinA reduces spasticity-associated pain, but further evidence is needed. This exploratory analysis pooled pain-relief data from six Phase 2 or 3 studies of incobotulinumtoxinA (four placebo-controlled studies) for treating upper limb spasticity in adults. Spasticity-associated pain was assessed at baseline and 4 weeks post incobotulinumtoxinA injection using the disability assessment scale (DAS) for pain. Only data for patients with pain at baseline were analysed. Overall, 544 (incobotulinumtoxinA, N = 415; placebo, N = 129) of 937 patients (58.1%) experienced pain at baseline. At Week 4, a significantly greater proportion of incobotulinumtoxinA- (52.1%) than placebo-treated patients (28.7%; Chi-square *p* < 0.0001) showed a response (≥1-point improvement in DAS pain score). In logistic regression analysis, incobotulinumtoxinA-treated patients were 2.6 times more likely to achieve this endpoint than placebo-treated patients. A significant difference between incobotulinumtoxinA and placebo was observed regardless of baseline pain severity. Additionally, 27.1% of incobotulinumtoxinA- versus 12.4% of placebo-treated patients reported complete pain relief at Week 4 (*p* = 0.0006). Pain relief increased with multiple injection cycles. To achieve patient-centred care, pain relief may be considered a treatment goal in adults with spasticity-associated pain regardless of pain severity. This study contributes to understanding the benefits of incobotulinumtoxinA in treating limb spasticity-associated pain.

## 1. Introduction

Spasticity of the upper and lower limbs following a stroke or as a consequence of other aetiologies (e.g., cerebral palsy, multiple sclerosis) can be a painful condition that limits patient mobility and quality of life, and impacts psychosocial well-being [1,2,3]. Pharmacologic options for the treatment of such spasticity include systemic antispastic agents, peripheral nerve block with neurolytic agents (e.g., phenol or alcohol) or intra-muscular injections with botulinum toxin type A (BoNT-A) [4,5,6,7,8,9].

The advantages of BoNT-A over systemic antispastics include local application and a selective action in the targeted spastic muscles, with better tolerability [6]. Three BoNT-A formulations are available in Europe and the USA for the treatment of limb spasticity: abobotulinumtoxinA, onabotulinumtoxinA and incobotulinumtoxinA. IncobotulinumtoxinA is a highly purified form of BoNT-A that is free from complexing proteins and may possibly demonstrate lower immunogenicity than other BoNT-A formulations containing these proteins, which are more likely to induce a host response than the neurotoxin itself [10,11]. Several clinical studies have demonstrated the efficacy and safety of incobotulinumtoxinA in the treatment of upper limb spasticity, showing that it improves muscle tone, spasticity-associated disability and quality of life with total injection doses ranging from 400 U up to 600 U [12,13,14,15,16,17,18].

The prevalence of pain among patients with limb spasticity varies widely from 46% to 74% depending on the patient population studied [19,20,21]. The efficacy of BoNT-A in reducing pain in patients with upper and lower limb spasticity has been investigated in clinical studies, either as a primary outcome [20,21,22,23,24,25] or a secondary outcome [12,14,15,17,26,27,28,29,30,31,32,33,34,35]. These studies have involved cohorts with sizes varying from 11 patients [29] to 487 patients [23]. The pain-relief results have been conflicting, but many patients with limb spasticity report pain relief following BoNT-A injections. However, in two meta-analyses that included an assessment of abobotulinumtoxinA in reducing limb spasticity-associated pain [36,37], this toxin was found to be no more effective than placebo in reducing limb spasticity-associated pain, although the quality of evidence was low.

This exploratory analysis pooled data explicitly on pain relief from six studies of incobotulinumtoxinA for the treatment of limb muscle spasticity in adults.

## 2. Results

A total of 937 patients participated in the six studies, of whom 544 (58.1%) experienced spasticity-associated pain (DAS pain score ≥1) at baseline and form the basis for this pooled analysis. Of the 544 patients, 83.8% (456/544) had mild or moderate pain, while 16.2% (88/544) had severe pain (Table 1). Of these patients, 415 received incobotulinumtoxinA and 129 received placebo. Supplementary Table S1 summarises treatment groups by individual study. Mean age was 56.4 ± 12.4 years and 60.5% were male. Baseline characteristics were generally similar between the two treatment groups, apart from the duration of spasticity and the proportion of BoNT-A-naïve subjects. In total, 97 patients (17.8%) took concomitant pain-relieving medication during the study, 81 (19.5%) in the incobotulinumtoxinA group and 16 (12.4%) in the placebo group; the difference was not statistically significant (Supplementary Table S2). Specifically, concomitant analgesics were taken by 13.0% and 8.5% of patients in the incobotulinumtoxinA and placebo groups, respectively, while concomitant anti-inflammatory/antirheumatic drugs were taken by 4.6% and 3.9% of patients, respectively. Use of these drugs was not statistically significantly different between the two treatment groups.

### 2.1. Frequency of Change in DAS Pain Score at Week 4

**Figure 1:**
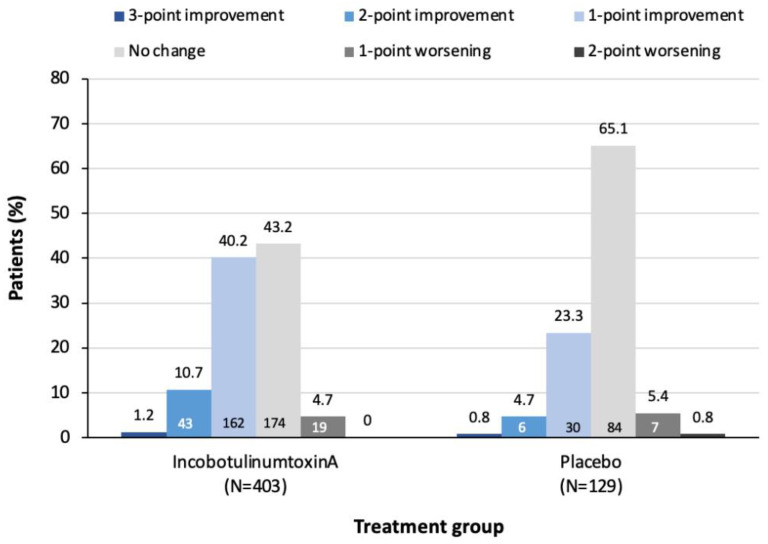
Proportion of patients with change in DAS pain score at Week 4, by treatment. Numbers in columns represent the number of patients in each category of change. DAS, disability assessment scale for pain.

A higher percentage of incobotulinumtoxinA-treated patients showed an improvement in DAS pain score of 1, 2 or 3 points at Week 4 compared to those treated with placebo. Additionally, patients treated with incobotulinumtoxinA were less likely to show no change in DAS pain score at Week 4 than placebo-treated patients (43.2% vs. 65.1%, respectively) (Figure 1).

### 2.2. Pain Response Rates at Week 4

Treatment with incobotulinumtoxinA produced a higher response rate (≥1-point improvement in DAS pain score) at Week 4 than treatment with placebo (52.1% vs. 28.7%, respectively). The difference in overall pain response rate between the incobotulinum-toxinA and placebo groups (23.4%; 95% CI: 14.2, 32.6) was statistically significant (Chi-square *p* < 0.0001). In logistic regression analysis, the OR for the difference in pain response rate between the two treatment groups was 2.62 (95% CI: 1.62, 4.24; *p* < 0.0001), reflecting that incobotulinumtoxinA-treated patients were 2.6 times more likely to show a ≥1-point improvement in DAS pain score at Week 4 than placebo-treated patients. In the sensitivity analysis of patients not taking concomitant pain-relief medication, the difference in pain response rate between incobotulinumtoxinA and placebo was still significantly in favour of incobotulinumtoxinA (difference in response rate between the two treatment groups 25.7%, 95% CI: 15.8, 35.6; Chi-square *p* < 0.0001) (Supplementary Table S3).

**Figure 2:**
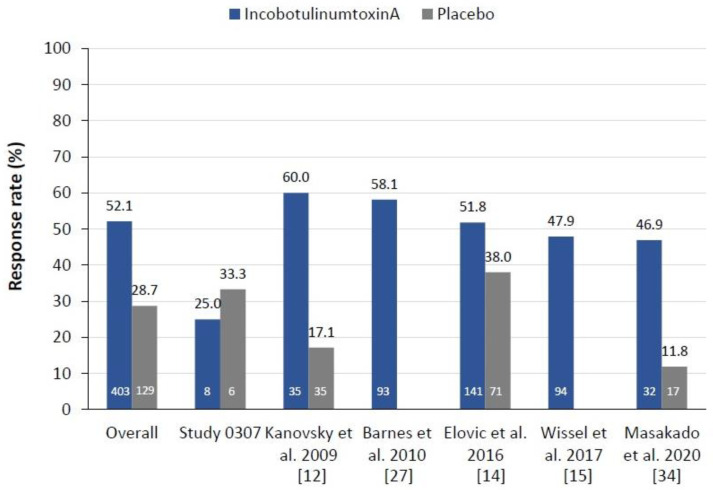
Response rates (≥1-point improvement in DAS pain score) at Week 4, by treatment and study. Numbers in columns represent the total number of patients experiencing pain in each treatment group. Note that the references cited for the individual studies do not report the data presented in the figure. DAS, disability assessment scale for pain.

A similar pattern of greater pain response rates with incobotulinumtoxinA versus placebo at Week 4 was seen across all completed individual studies, with incobotulinumtoxinA response rates generally ranging from 50% to 60%. The exception was the prematurely terminated study MRZ_60201_0307, in which two patients in each treatment group showed a response, resulting in a higher response rate in the placebo group (Figure 2).

**Figure 3:**
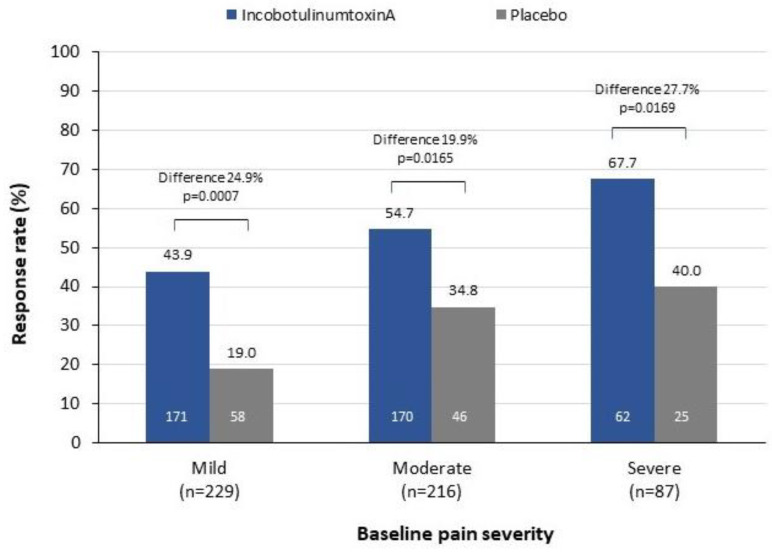
Difference in overall response rates (≥1-point improvement in DAS pain score) at Week 4, by treatment and baseline pain severity. Numbers in columns represent total number of patients in treatment group. *p*-values obtained from a Chi-Square comparison between treatment groups. DAS, disability assessment scale for pain.

Patients treated with incobotulinumtoxinA showed a significant improvement in overall pain response rate compared to placebo-treated patients, for those with mild, moderate or severe pain at baseline (Figure 3). The difference in pain response rate between incobotulinumtoxinA and placebo groups was 24.9% (95% CI: 12.4, 37.4, Chi-square *p* = 0.0007) for those with mild pain, 19.9% (95% CI: 4.3, 35.6; *p* = 0.0165) for those with moderate pain and 27.7% (95% CI: 5.3, 50.2; *p* = 0.0169) for those with severe pain.

### 2.3. Change in Pain Severity from Baseline to Week 4

**Figure 4:**
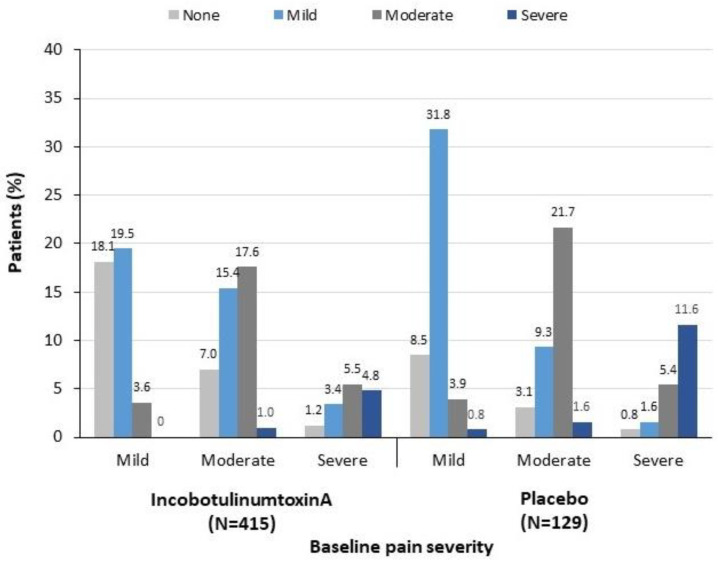
Change in pain severity from baseline to Week 4 following a single injection of incobotulinumtoxinA or placebo. The percentages shown at the top of each column are based on the total number of patients in the treatment group (N = 415 for incobotulinumtoxinA, N = 129 for placebo). Pain severity data at Week 4 were missing for 12 patients.

IncobotulinumtoxinA-treated patients were more likely than placebo-treated patients to report a lower level of pain severity at Week 4 compared with baseline, regardless of baseline pain severity (Figure 4). In addition, more incobotulinumtoxinA- than placebo-treated patients showed a change in DAS pain severity from baseline to Week 4 of ≥2 points (i.e., a change in pain severity from severe to mild or none, or from moderate to none) (Table 1 and Figure 4).

### 2.4. Complete Pain Relief at Week 4

Patients treated with incobotulinumtoxinA were significantly more likely to achieve complete pain relief than patients treated with placebo: at Week 4, 27.1% of incobotulinumtoxinA-treated patients (95% CI: 22.7, 31.4) versus 12.4% of placebo-treated patients (95% CI: 6.7, 18.1) experienced complete pain relief. The difference between the two treatment groups amounted to 14.6% (95% CI: 7.5, 21.8), which was statistically significant (Chi-square *p* = 0.0006).

### 2.5. Pain Response Rates and Proportion with Complete Pain Relief Following Multiple Injection Cycles

**Figure 5:**
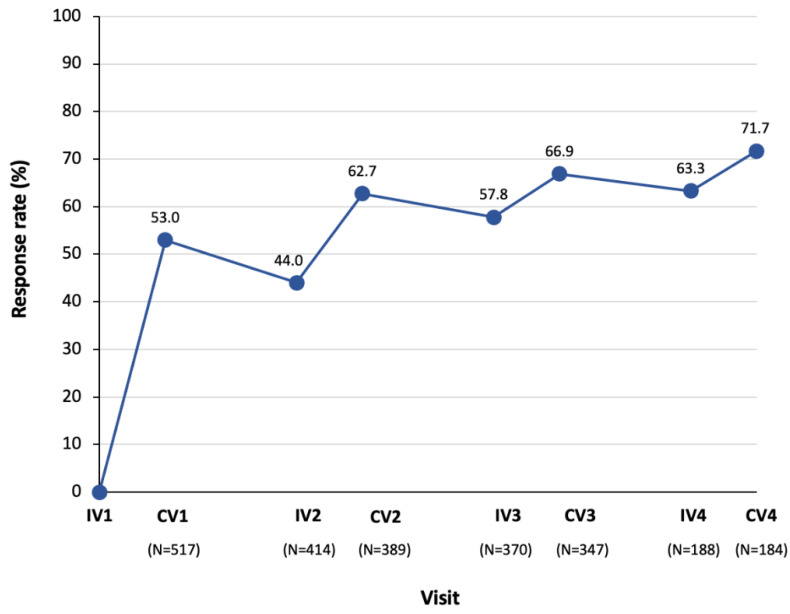
Response rates (≥1-point improvement in DAS pain score from baseline [Injection Visit 1]) with incobotulinumtoxinA over time. A control visit took place 4 weeks after the previous injection visit. The time between injections was 12–14 weeks. Only the first injection was placebo-controlled. As the placebo-treated patients received incobotulinumtoxinA in subsequent treatment cycles, their pain response rates to incobotulinumtoxinA have been included in injection Cycles 1 to 4 as appropriate, which explains the low sample size in injection Cycle 4. The N value given below each visit is the number of patients with data available for that visit. CV, control visit; DAS, disability assessment scale for pain; IV, injection visit.

**Figure 6:**
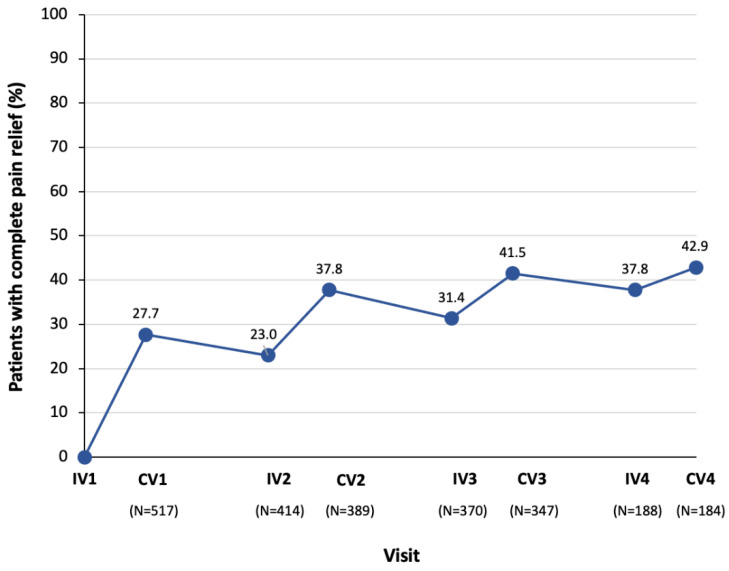
Proportion of patients with complete pain relief (measured using DAS for pain) with incobotulinumtoxinA over time. A control visit took place 4 weeks after the previous injection visit. The time between injections was 12–14 weeks. Only the first injection was placebo controlled. As the placebo-treated patients received incobotulinumtoxinA in subsequent treatment cycles, their pain response rates to incobotulinumtoxinA have been included in Injection Cycles 1 to 4 as appropriate, which explains the low sample size in Injection Cycle 4. The N value given below each visit is the number of patients with data available for that visit. CV, control visit; DAS, disability assessment Scale for pain; IV, injection visit.

Pain response rates with incobotulinumtoxinA increased over time, with 71.7% of patients showing a ≥1-point improvement in DAS pain score from baseline after four treatment cycles (Figure 5). Response rates were sustained and showed a cumulative effect throughout the study. The proportion of patients with complete pain relief also increased over time, with 42.9% experiencing complete pain relief after four treatment cycles (Figure 6).

## 3. Discussion

This pooled analysis of six studies, four of which were randomised, double-blind and placebo-controlled in design, showed that incobotulinumtoxinA significantly reduces pain compared with placebo in adult patients with upper limb spasticity-associated pain. These results confirm the pain-relieving benefits of incobotulinumtoxinA observed in the individual studies [12,14,15,27,34]. In this pooled population of patients with limb spasticity considered suitable for treatment with BoNT-A, approximately 60% of patients experienced pain at baseline. A significantly greater proportion of incobotulinumtoxinA- than placebo-treated patients achieved a ≥1-point improvement in DAS pain score at 4 weeks post injection, and incobotulinumtoxinA-treated patients were 2.6 times more likely to achieve such improvement than patients receiving placebo. In addition, patients treated with incobotulinumtoxinA were significantly more likely to achieve complete pain relief at Week 4 than patients treated with placebo. Moreover, response rates and the proportion of patients with complete pain relief increased over multiple incobotulinumtoxinA injection cycles. The pain-reducing benefits of incobotulinumtoxinA were observed in all pain severity groups, suggesting that pain relief may be considered an important treatment goal—regardless of pain severity level—to provide patient-centred care. Effective pain relief may also optimise gains in other treatment domains, such as improved mobility, active and passive function, independence in daily activities, sleep, quality of life and mental health.

Few studies have undertaken a pooled assessment of pain reduction in limb spasticity with other BoNT-A formulations. A meta-analysis of 10 randomised controlled trials (RCTs) of a single injection of BoNT-A (specific formulation not specified but studies published between 1989 and 2012 were reviewed) in upper or lower limb spasticity found that the quality of evidence for pain was low/very low (according to the Grading of Recommendations Assessment, Development and Evaluation approach; [38]). In addition, no significant pain-relieving effect in the upper or lower limbs 4–12 weeks post injection was found compared with placebo [36]. Similarly, a more recent meta-analysis of four RCTs of abobotulinumtoxinA for post-stroke upper limb spasticity showed that this formulation was no more effective than placebo in reducing spasticity-associated pain scores at 4–12 weeks post injection [37]. However, unlike our pooled analysis, neither of these meta-analyses focused specifically on patients with pain before BoNT-A treatment and the included studies used a variety of rating scales for assessing pain. These factors may contribute to the lack of a significant effect on spasticity-associated pain for BoNT-A versus placebo in the meta-analyses.

In this pooled analysis, the DAS pain response rate improved with each incobotulinumtoxinA treatment cycle. Interestingly, the re-emergence of pain between injection visits also diminished over time, possibly indicating a cumulative pain relief effect, which may translate into maintained pain control with long-term use. A post-hoc analysis of two studies of patients with upper limb spasticity who received injections of abobotulinumtoxin A into the shoulder muscles also found slightly greater improvements in DAS-rated pain with each injection cycle [21].

At the time when the studies included in this analysis were conducted, there was a lack of instruments specifically for measuring spasticity-associated pain. Most studies used non-specific pain scales, including numeric rating scales [20,23,25,32,39,40] or visual analogue scales (the most validated outcome measure in pain) [24,26,28,29,41] with score ranges of 0–10; a few used the DAS [21,24,30,31]. Since then, an instrument specifically for measuring spasticity-associated pain has been developed (the Questionnaire on Pain caused by Spasticity [QPS]; [42]), and this was used in later pediatric studies. In the six studies included in this pooled analysis, spasticity-associated pain was assessed using the DAS for pain [43]. The DAS is used primarily as a measure of functional disability across four domains of hygiene, dressing, limb position and pain. The pain domain covers different types of pain, including post-stroke neuropathic and central pain. Consequently, it can be difficult to quantify spasticity-associated pain. As the DAS for pain is scored on a 4-point scale, it has low granulation compared to the 11-point numeric rating scale and 100 mm visual analogue scale often used in pain studies. Thus, the significant differences in pain improvement with incobotulinumtoxinA versus placebo in our analyses are particularly noteworthy.

Often, studies using DAS assessments before and after BoNT-A treatment have focused on individual goal attainment, where patients—together with their physician—select one of the four DAS domains (hygiene, dressing, limb position, pain) as their individual principal therapeutic target. For example, in the study by Kanovsky et al. [12], which is included in our pooled analysis, although all four DAS domains were assessed, the primary therapeutic target chosen at the start of the study was dressing (for 40.5% patients), limb position (36.5%), hygiene (20.3%) and pain (3.4%). This suggests that few patients/clinicians consider decreased pain as the primary goal in spasticity management at the start of treatment. However, this study showed a high pain response rate to incobotulinumtoxinA treatment of 60% (see Figure 2) [12]. Another consideration is that the priority goal of such treatment may change with time and continuing treatment. In real-life clinical practice, patients with upper limb spasticity have multiple goals for treatment, including improvements in function, pain, mobility, sleep and quality of life [39,40,41], but successful pain management could positively contribute to the achievement of these goals.

Post-stroke pain is complex and heterogenous, with multiple contributory mechanisms [44]. Patients may in fact suffer from different types of pain, such as nociceptive and neuropathic pain [45,46]. Evidence suggests that BoNT-A has an analgesic effect against both types of pain [46,47,48]. The exact mechanisms of the pain-relieving effect of BoNT-A remain unclear, but are thought to be due to inhibition of peripheral and central sensitisation by retrograde axonal toxin transport, the inhibition of neuropeptides such as substance P, calcitonin gene-related peptide and glutamate, and deactivation of sodium channels [46,47,48,49]. The major benefits of BoNT-A over other analgesics are its sustained effect after a single injection—most likely due to the persistence of BoNT-A protease in neurons—and a low risk of adverse effects even after repeated injections [47]. Other analgesics (the most commonly used concomitant pain-relieving medication group in our pooled analysis), and opioids in particular, are associated with several potential long-term risks, including addiction and overdose as well as systemic side effects [50,51,52]. Treatment with BoNT-A (including incobotulinumtoxinA) may allow patients to reduce or stop their concomitant pain-relieving medications. Concomitant medication use at baseline was included in the present analysis, but any subsequent changes were not analysed; consequently, it is not known whether patients stopped or reduced concomitant pain medication at later timepoints. To detect meaningful correlations, frequent pain assessments and recordings of medication use (e.g., in diaries) would have been necessary, but this was beyond the scope of the studies included in the current analysis.

Providing pain relief is an important consideration for the holistic treatment of any disease to improve patient quality of life and avoid behavioural changes that may occur because of pain [53,54,55]. It appears that physicians are increasingly recognising the value of managing spasticity-associated pain with BoNT-A. In the Upper Limb International Spasticity (ULIS) studies reflecting real-life clinical practice, pain increased as the primary goal from 13% in ULIS-II to 25% in ULIS-III, and the proportion of patients who received injections for shoulder pain increased from 18% to 38% [56], suggesting that patients first consider functional benefit as the primary target before selecting pain, and this changes over time, or reflects a change in clinical practice.

Strengths of our analyses include the fact that they were performed in the largest patient cohort to date from mainly controlled studies; other studies of pain relief with BoNT-A in patients with upper and/or lower limb spasticity have involved population sizes of fewer than 500 patients [20,21,22,24,25,26,28,29,30,31,32]. In addition, the patient population came from a wide (international) geographic spread. Beneficial effects with incobotulinumtoxinA were observed across the studies involved in this analysis, in which multiple clinical patterns of the upper limb were treated [15]. Effective pain relief was achieved with incobotulinumtoxinA using a multipattern treatment approach, together with its well-established safety profile and low immunogenicity profile [11]. Other strengths include that 42% of patients from the six studies had no pain at baseline, and were therefore, not included in the pooled analyses, thereby avoiding a possible ‘dilution effect’ from data from patients with no pain at baseline; a standard measure of pain (DAS) was used across the six studies included in our analyses; data were obtained over multiple incobotulinumtoxinA injection cycles; and the analyses were performed on a background of stable antispastic medications (centrally acting muscle relaxants, benzodiazepine) and physical/occupational therapy.

Limitations of our analyses include potential bias due to different patient numbers from the different studies used in the pooled analyses. For example, the PURE study [14] contributed around 40% of the patients analysed, whereas study MRZ_60201_0307 contributed only 3%. In addition, the aetiology of spasticity was mixed, although most patients (95%) had post-stroke spasticity. As spasticity-associated pain can change over time, it is possible that some patients with no pain at baseline developed pain during the study. Finally, although not validated, it was reasonably assumed that a ≥1-point improvement in DAS score for pain (change from severe to moderate or from moderate to mild pain) is clinically relevant.

This analysis provides additional evidence to support the use of incobotulinumtoxinA for reducing limb spasticity-associated pain in adults, which was seen by 4 weeks post injection, and warrants further controlled studies to confirm the pain-relieving benefits of BoNT-As in post-stroke spasticity and to investigate potential earlier onset of effects. The results of this analysis are relevant for clinicians treating this patient population by highlighting the benefits of incobotulinumtoxinA as an option for pain relief at all levels of pain severity.

## 4. Conclusions

This pooled analysis showed that incobotulinumtoxinA is significantly more effective than placebo in reducing limb spasticity-associated pain at 4 weeks post injection in adults. This is the largest patient cohort analysed to date in this setting, providing additional evidence to support the use of incobotulinumtoxinA for pain relief. Almost 60% of adults in this analysis experienced limb spasticity-associated pain at baseline, and approximately 20% of these patients experienced severe pain. The pain-relieving effects of incobotulinumtoxinA increased as the number of treatment cycles increased and were observed regardless of pain severity. This suggests that pain relief may be considered an important treatment goal in all patients with spasticity-associated pain to provide patient-centred care. IncobotulinumtoxinA may represent an important local treatment option to achieve better outcomes for patients with limb spasticity by significantly reducing associated pain.

## 5. Materials and Methods

### 5.1. Studies Included in the Analyses

Analyses were based on pooled results from six prospective, multicentre, Phase 2 or 3 studies of incobotulinumtoxinA (Xeomin^®^; Merz Pharmaceuticals GmbH, Frankfurt, Germany) in the treatment of upper (five studies: MRZ_60201_0307); [12,14,27,34]) and/or lower (one study: [15]) limb muscle spasticity in adults. The studies were part of the clinical development programme for incobotulinumtoxinA and were performed across multiple countries and continents worldwide, including Europe, Canada, USA, Japan, India and Russia. Of the six studies, four were randomised, double-blind and placebo-controlled, while two evaluated different incobotulinumtoxinA doses/dilutions and were not placebo controlled (Table 2).

In all studies, adult patients aged ≥18 years who had not received BoNT-A injections within at least 4 months of screening received incobotulinumtoxinA injections as appropriate for their condition. Each injection was followed by 12 weeks of observation and assessment. In studies where patients received more than one incobotulinumtoxinA injection, the time between injections was 12–14 weeks. The spasticity patterns treated in each study are shown in Table 2.

Antispastic medications (centrally acting muscle relaxants, benzodiazepine) and physical/occupational therapy were allowed during the studies, provided they were stable in the 2–4 weeks prior to screening. No changes to such treatments were allowed during the study periods. Peripheral muscle relaxants were not allowed. The use of analgesic medication was permitted during the studies and recorded under concomitant medication use.

The intensity of pain or discomfort associated with limb spasticity was evaluated by the investigator based on patient questioning at baseline and at 4 weeks post incobotulinumtoxinA injection using the disability assessment scale (DAS) for pain. This scale measures the intensity of pain or discomfort associated with muscle spasticity on a scale of 0 (no pain) to 3 (severe pain) [43]. In one study [27], DAS pain was measured as the primary outcome if pain had been chosen by the patient as the primary therapeutic target (see footnote to Table 2); in all other studies, DAS pain was evaluated as a secondary outcome. In all studies, muscle tone in the affected/treated muscle groups was measured (either as a primary or secondary outcome) at baseline and at 4 weeks post incobotulinumtoxinA injection using the Ashworth or modified Ashworth scale [57,58] (Table 2).

All studies were conducted in accordance with the Declaration of Helsinki and Good Clinical Practice, and were approved by the ethics committee for each participating site. All patients provided written informed consent prior to study participation.

### 5.2. Analyses

This pooled analysis evaluated data only for patients with spasticity-associated pain at baseline based on a DAS pain score of ≥1. For the analyses involving a comparison with placebo, only data from the first injection cycle were analysed; further treatment cycles were not placebo controlled. Data analysed at Week 4 following an incobotulinumtoxinA injection included the frequency of change in DAS pain score for incobotulinumtoxinA- and placebo-treated patients, and the percentage of incobotulinumtoxinA- and placebo-treated patients who responded to treatment, where a response was defined as a ≥1-point improvement in DAS pain score from baseline (Injection Visit 1), as used in other studies [30,60]. The difference in response rates between incobotulinumtoxinA and placebo groups overall and by severity of baseline pain (mild, moderate, severe) was evaluated using a Chi-square test. The difference in response rates between the two treatment groups was also analysed using logistic regression analysis (presented as an odds ratio [OR] and 95% Wald confidence interval [CI]), with the baseline DAS pain score and study as covariates. The percentage of patients achieving complete pain relief (DAS pain score = 0) was also evaluated, and the difference between the two treatment groups analysed using a Chi-square test. In addition, response rates and the percentage of patients with complete pain relief over time based on all injection cycles relative to baseline (injection Visit 1) were analysed at injection visits (1, 3, 5 and 7) and 4 weeks (±3 days) after each injection (Visits 2, 4, 6 and 8). Finally, the difference in concomitant pain medication use between incobotulinumtoxinA- and placebo-treated patients during the study was evaluated using Fisher’s exact test. A sensitivity analysis was performed that included only patients not taking concomitant pain-relieving medication, to confirm the lack of influence of these drugs on pain response rates. The difference in response rates between the two treatment groups was analysed using a Chi-square test. All analyses were based on observed cases (i.e., there was no strategy for missing data as few pain-relief data were missing [*n* = 12]), and were performed using Statistical Analysis Software (SAS) 9.4 (SAS Institute Inc., Cary, NC, USA). A *p*-value of ≤0.05 was considered statistically significant.

## Figures and Tables

**Table 1:** Baseline characteristics for patients with pain at baseline.

Characteristic	IncobotulinumtoxinA (N = 415)	Placebo (N = 129)	Total (N = 544)
Age (years)	56.5 ± 12.9	56.2 ± 10.6	56.4 ± 12.4
Age group			
18–50 years	124 (29.9)	38 (29.5)	162 (29.8)
51–65 years	180 (43.4)	64 (49.6)	244 (44.9)
≥66 years	111 (26.8)	27 (20.9)	138 (25.4)
Male	249 (60.0)	80 (62.0)	329 (60.5)
Ethnicity			
White	324 (78.1)	99 (76.7)	423 (77.8)
Asian	57 (13.7)	29 (22.5)	86 (15.8)
Black or African American	9 (2.2)	1 (0.8)	10 (1.8)
Other	4 (1.0)	0	4 (0.7)
Missing	21 (5.1)	0	21 (3.9)
Height (cm)	167.8 ± 9.4 ^a^	168.8 ± 8.3	168.0 ± 9.1
Weight (kg)	75.7 ± 15.0 ^b^	74.5 ± 15.4	75.4 ± 15.1
Duration of spasticity (time since diagnosis, years)	5.4 ± 6.7 ^c^	3.3 ± 4.8	4.9 ± 6.3
Aetiology of spasticity			
Stroke	389 (93.7)	128 (99.2)	517 (95.0)
Brain injury	10 (2.4)	0	10 (1.8)
Infantile cerebral palsy	5 (1.2)	0	5 (0.9)
Multiple sclerosis	0	1 (0.8)	1 (0.2)
Other	11 (2.7)	0	11 (2.0)
Baseline DAS pain score			
1 = Mild	177 (42.7)	58 (45.0)	235 (43.2)
2 = Moderate	175 (42.2)	46 (35.7)	221 (40.6)
3 = Severe	63 (15.2)	25 (19.4)	88 (16.2)
BoNT-A naïve	202 (48.7)	94 (72.9)	296 (54.4)
Patients taking concomitant pain-relieving medication	81 (19.5)	16 (12.4)	97 (17.8)

Results are presented as mean ± standard deviation or n (%). ^a^ Data were missing from one patient for height. ^b^ Data were missing from two patients for weight. ^c^ Data were missing from five patients for duration of spasticity. BoNT-A, botulinum toxin type A; DAS, disability assessment scale for pain.

**Table 2:** Details of the six studies included in the pooled analyses.

Study Name, NCT Number and Reference(s)	Phase	Study Design and Objective	Patients and Indication	Treatments	Clinical Patterns Treated	Primary Outcome
MRZ_60201_0307	2	Prospective, randomised, double-blind, placebo-controlled, parallel-group, multicentre pilot study (12 weeks) to investigate the efficacy and safety of incobotulinumtoxinA in the treatment of pain in upper limb spasticity	N = 14 adults with pain caused by upper limb spasticity due to multiple aetiologies ^a^	(1) IncobotulinumtoxinA, up to 400 U (2) Placebo	Flexed elbow Clenched fist Flexed wrist Thumb in palm Pronated forearm Adducted/ internally rotated shoulder Intrinsic plus hand	Mean evening pain intensity measured using the 11-point box scale ^b^
MRZ_60201_0410 NCT00432666 Kanovsky et al. 2009 [12] Kanovsky et al. 2011 [13]	3	Prospective, randomised, double-blind, placebo-controlled, parallel-group, multicentre trial (20 weeks) with an open-label extension period (69 weeks) to investigate the efficacy and safety of incobotulinumtoxinA in the treatment of post-stroke upper limb spasticity	N = 148 adults with post-stroke upper limb spasticity	(1) IncobotulinumtoxinA, 170–400 U (2) Placebo	Flexed elbow Clenched fist Flexed wrist Thumb in palm Pronated forearm	Muscle tone response rate (≥1-point improvement in Ashworth Scale score) at Week 4
MRZ_60201_0607 NCT00465738 Barnes et al. 2010 [27]	3	Prospective, randomised, observer-blind, parallel-group, multicentre trial (20 weeks) to assess the efficacy and safety of two different dilutions of incobotulinumtoxinA in patients with upper limb spasticity	N = 192 adults with stable upper limb spasticity of diverse aetiology	(1) IncobotulinumtoxinA, 400 U in 8 mL (2) IncobotulinumtoxinA, 400 U in 20 mL	Flexed elbow Clenched fist Flexed wrist Thumb in palm Pronated forearm Intrinsic plus hand	DAS response rate (≥1-point improvement) at Week 4 ^c^
PURE MRZ_60201_SP3001 NCT01392300 Elovic et al. 2016 [14] Marciniak et al. 2019 [59] and 2020 [18]	3	Prospective, randomised, double-blind, placebo-controlled, parallel-group, multicentre study (12 weeks) with an open-label extension period (36 weeks) to investigate the efficacy and safety of incobotulinumtoxinA in the treatment of post-stroke upper limb spasticity	N = 317 adults with post-stroke upper limb spasticity	(1) IncobotulinumtoxinA, 400 U (2) Placebo	Flexed elbow Clenched fist Flexed wrist Thumb in palm Pronated forearm ^d^	Change in muscle tone from baseline to Week 4, measured using the Ashworth Scale
TOWER MRZ_60201_3053 NCT01603459 Wissel et al. 2017 [15]	3	Prospective, non-randomised, open-label, single-arm, multicentre dose-titration study (48 weeks) to investigate the safety and efficacy of incobotulinumtoxinA in subjects requiring doses of 800 U during the course of the study for the treatment of upper and lower limb spasticity	N = 155 adults with chronic upper and lower limb spasticity of the same body side due to cerebral causes	(1) IncobotulinumtoxinA, 400 U (2) IncobotulinumtoxinA, 600 U (3) IncobotulinumtoxinA, 600–800 U	Upper limb Flexed elbow Clenched fist Flexed wrist Thumb in palm Pronated forearm Extended elbow Internally rotated/extended/adducted shoulder Lower limb Flexed hip Internally rotated hip	Safety
J-PURE MRZ_60201_3099 CTI-153029 (Japanese Clinical Trials Registry) Masakado et al. 2020 [34]	3	Prospective, randomised, double-blind, placebo-controlled, parallel-group, multicentre study (52 weeks in total), with an open-label lead-in tolerability period (1 week), a main study period (12 weeks) and an open-label extension period (32–40 weeks), to investigate the efficacy and safety of two different doses of incobotulinumtoxinA in the treatment of post-stroke upper limb spasticity	N = 111 adults with post-stroke upper limb spasticity	(1) IncobotulinumtoxinA, 400 U (2) Placebo, 400 U (3) IncobotulinumtoxinA, 250 U (4) Placebo, 250 U	Flexed elbow and pronated forearm Clenched fist Flexed wrist Thumb in palm	Change in muscle tone from baseline to Week 4, measured using the modified Ashworth Scale

^a^ Study stopped early due to low recruitment. ^b^ These pain data are not included in the current pooled analysis as they were not measured in the other studies. ^c^ In this study, patients chose one of four domains of the DAS (dressing, limb position, pain, hygiene) as the primary therapeutic target; this was limb position in 63%, dressing in 24%, pain in 6% and hygiene in 8% of patients. ^d^ In PURE, the primary target clinical pattern treated included flexed elbow, flexed wrist or clenched fist at predefined fixed doses. Other clinical patterns could be treated with the remainder of the total dose as medically indicated. DAS, disability assessment scale for pain.

## Data Availability

Data are not publicly available.

## Supplementary Materials

The following are available online at https://www.mdpi.com/article/10.3390/toxins13120887/s1, Table S1: patients with pain at baseline who contributed data for the pooled analyses, by study, Table S2: use of concomitant pain-relieving medication throughout the study in patients with pain at baseline, Table S3: response rates (≥1-point improvement in DAS pain score) at Week 4 by treatment in the sensitivity analysis of patients not taking concomitant pain-relieving medication during the study.
