# Supplementary material for: Pain Reduction in Adults with Limb Spasticity Following Treatment with IncobotulinumtoxinA: A Pooled Analysis
Source: Toxins (Basel). 2021 Dec 11;13(12):887. doi: 10.3390/toxins13120887 (PMC8704318; doi:10.3390/toxins13120887)
Supplement: Supplementary file 1 [file toxins-13-00887-s001.zip › toxins-1463098-SU-12.9-12.20.pdf]

# Supplementary Materials: Pain Reduction in Adults with Limb Spasticity following Treatment with IncobotulinumtoxinA: A Pooled Analysis

**Jörg Wissel, Alexandre Camões-Barbosa, Georg Comes, Michael Althaus Astrid Scheschonka and David M. Simpson**

**Table S1**. Patients with pain at baseline who contributed data for the pooled analyses, by study.

| Study and primary reference | IncobotulinumtoxinA (*N* = 415) | Placebo (*N* = 129) | Total (*N* = 544) |
|---|---|---|---|
| MRZ_60201_0307 | 8 | 6 | 14 |
| MRZ_60201_0410<br>NCT00432666 (Kanovsky et al. 2009) [12] | 35 | 35 | 70 |
| MRZ_60201_0607<br>NCT00465738 (Barnes et al. 2010) [27] | 94 | – | 94 |
| MRZ_60201_SP3001<br>NCT01392300 (Elovic et al. 2016) [14] | 144 | 71 | 215 |
| MRZ_60201_3053<br>NCT01603459 (Wissel et al. 2017) [15] | 102 | – | 102 |
| MRZ_60201_3099<br>CTI-153029 (Masakado et al. 2020) [34] | 32 | 17 | 49 |

**Table S2.** Use of concomitant pain-relieving medication throughout the study in patients with pain at baseline.

| ATC drug class level 2 | IncobotulinumtoxinA ($N$ = 415) | Placebo ($N$ = 129) | Total ($N$ = 544) |
|---|---|---|---|
| Patients taking concomitant pain-relieving medication | 81 (19.5) | 16 (12.4) | 97 (17.8) |
| Analgesics | 54 (13.0) | 11 (8.5) | 65 (11.9) |
| Anti-inflammatory/ antirheumatic drugs | 19 (4.6) | 5 (3.9) | 24 (4.4) |
| Psychoanaleptics | 6 (1.4) | 0 | 6 (1.1) |
| Topical products for joint and muscular pain | 5 (1.2) | 2 (1.6) | 7 (1.3) |
| Anaesthetics | 2 (0.5) | 0 | 2 (0.4) |
| Muscle relaxants | 0 | 2 (1.6) | 2 (0.4) |
| Antiepileptics | 1 (0.2) | 0 | 1 (0.2) |

Results are presented as $n$ (%).
Differences between the two treatment groups were not statistically significant (Fisher's exact test $p>0.05$).
ATC, Anatomical, Therapeutic, Chemical.

**Table S3**. Response rates (≥1-point improvement in DAS pain score) at week 4 by treatment in the sensitivity analysis of patients not taking concomitant pain-relieving medication during the study.

| Responder | Treatment group | | | Difference | |
|---|---|---|---|---|---|
| | IncobotulinumtoxinA (*N* = 324) | Placebo (*N* = 113) | Total (*N* =437) | Response rate (%) | p-value (95% CI)[a] |
| Yes | 175 (54.0) | 32 (28.3) | 207 (47.4) | 25.7 | <0.0001 (15.8, 35.6) |
| No | 149 (46.0) | 81 (71.7) | 230 (52.6) | | |

Data are presented as *n* (%).
[a]p-value from a Chi Square test.
CI, confidence interval; DAS, Disability Assessment Scale for pain.
